# Deep brain stimulation for obsessive-compulsive disorder: Why phenotype matters

**DOI:** 10.1016/j.neurot.2026.e01104

**Published:** 2026-11-04

**Authors:** Akihiro Takamiya, Ki Sueng Choi, Allison Waters, Helen Mayberg, Martijn Figee

**Affiliations:** a Nash Family Center for Advanced Circuitry Therapeutics, Icahn School of Medicine at Mount Sinai, New York, NY, USA; b Department of Psychiatry, Icahn School of Medicine at Mount Sinai, New York, NY, USA; c Department of Radiology, Icahn School of Medicine at Mount Sinai, New York, NY, USA; d Department of Neurology, Icahn School of Medicine at Mount Sinai, New York, NY, USA

**Keywords:** OCD, DBS, ALIC, MFB

The history of neurosurgical treatment for obsessive-compulsive disorder (OCD) has closely followed evolving concepts of how psychiatric symptoms arise from dysfunctional brain circuits. Early psychosurgical procedures rested on the premise that pathological interactions between frontal cortical and subcortical regions could be disrupted by surgical lesions. With the development of stereotactic functional neurosurgery, procedures such as anterior capsulotomy allowed increasingly precise, anatomically defined interventions. Deep brain stimulation (DBS) subsequently emerged as an adjustable and reversible alternative to ablative surgery. In 1999, Nuttin and colleagues [1] reported chronic stimulation of the anterior limb of the internal capsule (ALIC) as an alternative to bilateral capsulotomy in patients with severe, treatment-refractory OCD. Over the subsequent decades, multiple targets have been explored, including the ALIC, ventral capsule/ventral striatum (VC/VS), nucleus accumbens, bed nucleus of the stria terminalis, subthalamic nucleus (STN), and, more recently, the superolateral medial forebrain bundle (slMFB). Across targets and techniques, approximately two thirds of patients meet conventional response criteria at last follow-up [2], and active stimulation has shown a significant benefit over sham stimulation [3]. Nevertheless, a substantial proportion of patients derive only partial or limited benefit. Understanding the sources of this variability has, therefore, become a central challenge for the field.

One obvious source of variability is anatomy. Many psychiatric DBS targets encompass complex white-matter systems rather than discrete nuclei, and the trajectories of these pathways vary across individuals. DBS has, therefore, progressively shifted from targeting coordinates toward identifying and selectively engaging therapeutic circuits. This evolution has been especially prominent in subcallosal cingulate DBS for depression, in which prospective tractography-guided targeting was developed from a responder-derived white-matter “connectome blueprint" [4], [5] and is now being evaluated in the multicenter, randomized, sham-controlled TRANSCEND trial (NCT06423430) [6]. A similar strategy has since been applied to OCD. Our group developed a patient-specific ALIC targeting approach based on a common responder map involving connections to the ventromedial prefrontal cortex (vmPFC)/orbitofrontal cortex (OFC), ventrolateral prefrontal cortex (vlPFC), and midbrain [7]. Other work has further deconstructed the pathways engaged by different OCD targets. For example, Coenen and colleagues [8] recently reported that slMFB and anteromedial STN stimulation engage overlapping but also distinct OCD-related subnetworks, challenging the notion that all effective OCD DBS sites act through a single common fiber pathway. Whether increasingly precise anatomical targeting will consistently translate into higher and more predictable response rates across OCD populations remains to be established [9]. Nevertheless, the direction of the field is clear: we can define where and what we stimulate with increasing precision. Anatomical precision, however, was never expected to be sufficient on its own. As previously emphasized, even with increasingly refined connectomic targeting, patient heterogeneity remains a central obstacle to circuit-specific treatment [10].

The study by Czornik and colleagues [11] in this issue of Neurotherapeutics raises the logical next question: even if we can stimulate the intended circuit precisely, are we stimulating the right circuit for the individual patient? In 14 patients receiving DBS consistently targeted at the same slMFB white matter fibers, the authors examined changes across symptom domains of the Obsessive-Compulsive Inventory-Revised (OCI-R) and whether baseline symptom profiles predicted treatment response. The obsessing domain showed the largest numerical improvement, and a greater baseline proportion of obsessing symptoms was associated with greater subsequent improvement on the Yale-Brown Obsessive-Compulsive Scale (Y-BOCS). This suggests that DBS at the slMFB, a key component of the brain's reward and motivation circuitry, could primarily attenuate the affective salience of obsessions, which may be different from OCD targets that are more primarily affecting compulsivity such as the nearby STN [12] or specific ALIC connections [7]. The study, therefore, advances an important concept: precision DBS may require not only anatomical precision but also better characterization of the patient. At the same time, these findings should be regarded as hypothesis-generating. The cohort size was modest, the predictor analysis was exploratory, and the baseline-adjusted model did not show significant time-by-subscale interaction, so differential improvement across symptom domains could not be statistically established. More fundamentally, it remains uncertain what an “obsessing" phenotype represents biologically.

The OCI-R obsessing subscale used by Czornik and colleagues [11] reflects intrusive, unwanted, and difficult-to-control thoughts regardless of OCD symptom theme. Traditional OCD classifications have largely emphasized symptom themes, such as contamination/cleaning, taboo thoughts (e.g., aggressive, sexual, or religious thoughts), or symmetry/ordering. However, similar symptom themes may arise from different underlying motivations. A compulsion may be performed to prevent a feared outcome, to resolve uncertainty, or because something feels incomplete or “not just right.” The “classic” model of OCD has largely emphasized fear-based symptoms, in which obsessions signal potential harm and compulsions are performed to prevent or neutralize that feared consequence. However, this framework may not adequately capture sensory-driven symptoms, in which repetitive behaviors are motivated by uncomfortable bodily sensations, urges, or feelings of incompleteness rather than an explicit feared consequence [13]. A clinically useful phenotype may, therefore, need to specify not only the theme of a patient's symptoms but also the motivation behind them.

Contamination/cleaning symptoms provide an intuitive example. Two patients may both wash repeatedly, yet one may do so to prevent a feared harmful consequence such as contamination or illness, while the other may wash to neutralize disgust or sensory discomfort. At the level of symptom theme, both have contamination/cleaning symptoms; at the level of motivation, they may be fundamentally different [14]. Preliminary DBS data suggest that such differences may matter clinically. In a small retrospective cohort, nine of ten patients with predominantly harm-avoidant (fear-based) contamination symptoms met response criteria after VC/VS DBS, compared with one of four patients with predominantly disgust-driven symptoms [15]. This differential response suggests the possibility that harm-avoidance and disgust may involve partly distinct neural circuits.

Czornik and colleagues [11] rightly point toward phenotype-guided neuromodulation. The purpose of phenotyping, however, should not be merely to identify patients who are likely to respond poorly to an existing intervention. A useful phenotype should generate a mechanistic and ultimately therapeutic hypothesis. If distinct motivations of compulsivity, such as fear/harm-avoidance, sensory phenomena/incompleteness, or uncertainty, map onto distinguishable brain-behavioral subtypes, they may point toward different therapeutic circuits. For DBS, this could inform which pathways should be engaged, which contacts should be selected, and how stimulation should be programmed. In other patients, the relevant circuit may contain a cortical node more accessible to complementary approaches such as transcranial magnetic stimulation or focused ultrasound. Individualized stimulation of the postcentral gyrus has already been explored as a means of targeting sensory urges in OCD [16]. Moving from association to intervention will, therefore, require prospective studies that explicitly test the sequence from clinical phenotype to biology to therapeutic target.

The next stage of precision neuromodulation may, therefore, require integrating two forms of precision. Anatomical precision for modulating the intended pathway and phenotypic precision for modulating the pathway most relevant to the individual patient. More than 25 years after DBS was introduced as a reversible alternative to capsulotomy, increasingly sophisticated methods allow us to define where to stimulate. The next challenge is to determine which circuit matters for whom and why.

## Author contributions

Akihiro Takamiya wrote first draft.

All authors helped conceptualizing and editing the manuscript.

## Funding

This work was supported by a grant from the National Institute of Mental Health to M.F. and K.S.C. (R01 MH123542).

## Declaration of competing interest

The authors declare the following financial interests/personal relationships which may be considered as potential competing interests:

Martijn Figee reports financial support was provided by Abbott. Martijn Figee reports financial support was provided by Medtronic. If there are other authors, they declare that they have no known competing financial interests or personal relationships that could have appeared to influence the work reported in this paper. Dr. Choi has served as a consultant for Abbott. Dr. Figee has served as a consultant for Abbott and Medtronic. Dr. Mayberg has served as a consultant for Abbott Neuromodulation, Abb-Vie, BlackrockNeuro, and NextSense; she receives intellectual property licensing fees from Abbott Neuromodulation; and she has served on scientific advisory boards for the Brain and Behavior Research Foundation and the Klingenstein Foundation. The other authors report no financial relationships with commercial interests.
